# Obesity inhibits the osteogenic differentiation of human adipose-derived stem cells

**DOI:** 10.1186/s12967-016-0776-1

**Published:** 2016-01-27

**Authors:** Amy L. Strong, Ryan S. Hunter, Robert B. Jones, Annie C. Bowles, Maria F. Dutreil, Dina Gaupp, Daniel J. Hayes, Jeffrey M. Gimble, Benjamin Levi, Margaret A. McNulty, Bruce A. Bunnell

**Affiliations:** Center for Stem Cell Research and Regenerative Medicine, Tulane University School of Medicine, 1430 Tulane Avenue, New Orleans, LA 70112 USA; Department of Biological and Agricultural Engineering, Louisiana State University, Baton Rouge, LA 70803 USA; LaCell LLC, New Orleans, LA 70112 USA; Department of Surgery, Tulane University School of Medicine, New Orleans, LA 70112 USA; Department of Surgery, Division of Plastic Surgery, University of Michigan, Ann Arbor, MI USA; Department of Comparative Biomedical Sciences, School of Veterinary Medicine, Louisiana State University, Baton Rouge, LA 70803 USA; Department of Pharmacology, Tulane University School of Medicine, New Orleans, LA 70112 USA

**Keywords:** ASCs, Adipose derived stromal/stem cells, Osteogenesis, Obesity, BMI

## Abstract

**Background:**

Craniomaxillofacial defects secondary to trauma, tumor resection, or congenital malformations are frequent unmet challenges, due to suboptimal alloplastic options and limited autologous tissues such as bone. Significant advances have been made in the application of adipose-derived stem/stromal cells (ASCs) in the pre-clinical and clinical settings as a cell source for tissue engineering approaches. To fully realize the translational potential of ASCs, the identification of optimal donors for ASCs will ensure the successful implementation of these cells for tissue engineering approaches. In the current study, the impact of obesity on the osteogenic differentiation of ASCs was investigated.

**Methods:**

ASCs isolated from lean donors (body mass index <25; lnASCs) and obese donors (body mass index >30; obASCs) were induced with osteogenic differentiation medium as monolayers in an estrogen-depleted culture system and on three-dimensional scaffolds. Critical size calvarial defects were generated in male nude mice and treated with scaffolds implanted with lnASCs or obASCs.

**Results:**

lnASCs demonstrated enhanced osteogenic differentiation in monolayer culture system, on three-dimensional scaffolds, and for the treatment of calvarial defects, whereas obASCs were unable to induce similar levels of osteogenic differentiation in vitro and in vivo. Gene expression analysis of lnASCs and obASCs during osteogenic differentiation demonstrated higher levels of osteogenic genes in lnASCs compared to obASCs.

**Conclusion:**

Collectively, these results indicate that obesity reduces the osteogenic differentiation capacity of ASCs such that they may have a limited suitability as a cell source for tissue engineering.

**Electronic supplementary material:**

The online version of this article (doi:10.1186/s12967-016-0776-1) contains supplementary material, which is available to authorized users.

## Background

Bone defects in the craniomaxillofacial skeleton can occur as a result of congenital defects or acquired injuries secondary to trauma, surgery, infection, and cancer. Current options for treatment include autologous bone grafts and microvascular free flaps. However, drawbacks of these procedures include limited donor tissue, donor site morbidity, unpredictable resorption rate, and high infection rates of both the donor and recipient sites [1–4]. Furthermore, in the osteoporotic patient, these bone grafts are likely to be osteoporotic as well, limiting their ability to heal fracture sites. Additionally, alloplastic materials, though temporizing, often are complicated by breakdown, resorption, or infection. Therefore, reconstruction of these bone defects remains a significant challenge with high morbidity.

Alternative strategies have recently utilized tissue-engineering approaches to supplement biodegradable scaffolds with adipose-derived stromal/stem cells (ASCs) and shown significant promise in treating craniomaxillofacial defects. ASCs are self-renewing, multipotent stromal cells with the capacity to differentiate into osteoblasts [5–7]. ASCs secrete an abundance of growth factors that assist in angiogenesis and bone regeneration [8, 9]. Animal experiments have shown the feasibility of healing critical size calvarial and mandibular defects with ASC seeded scaffolds [10–13]. Similar efficacy has been demonstrated with ASC seeded scaffolds for the treatment of femoral defects and vertebral defects [14, 15]. Human clinical trials have demonstrated the regenerative potential of ASC seeded scaffolds to aid in craniomaxillofacial hard tissue reconstruction [16, 17]. Implantation of restorable scaffold material seeded with ASCs demonstrated successful integration of the constructs with the surrounding skeleton [16, 17]. The bony defects demonstrated significant remodeling that likely contributed to the long-term engraftment of the constructs and regeneration of the tissue.

With the growing interest and promise of novel tissue engineering approaches utilizing ASCs as the cell source, identifying the optimal donor and factors that may alter ASC biology is pertinent to the success of this method. Alterations to ASC biology may dramatically reduce the attachment of the ASCs to the scaffold and reduce the efficacy of the construct for bone healing. Recently, studies have shown that ASCs isolated from older donors displayed reduced viability, self-renewal capacity, proliferation, and differentiation potential, compared to ASCs isolated from young donors [18, 19]. The effects of aging on ASC osteogenesis translated to reduced formation of osteoblast-like cells on scaffolds [19]. ASCs have also been shown to promote the survival of endothelial cells and coordinate with the local environment to form vascular networks to assist in healing [20–22]. However, the angiogenic effects of ASCs are compromised with advanced age and following continuous exposure to chronic diseases, such as coronary artery disease [23–25]. Together, these studies demonstrate that exposure to chronic inflammation may impact stem cell function, and these alterations may be detrimental for the success of tissue engineering approaches.

Obesity is associated with low-grade chronic inflammation and is characterized by excess lipid accumulation and overproduction of inflammatory cytokines associated with the adipocyte hyperplasia and hypertrophy [26, 27]. Studies have shown that ASCs isolated from obese donor have loss of stemness markers and increased expression of inflammatory cytokines [28]. Furthermore, the recipient site of ASC implantation in diabetic patients is often compromised with regards to its angiogenic and osteogenic niche. Additional studies have postulated that the reason ASCs isolated from obese donors have altered biological properties is due to their close proximity to the inflammatory microenvironment [29, 30]. Nevertheless, it remains to be determined whether obesity influences the osteogenic differentiation potential ASCs on biodegradable scaffolds and whether these effects would impede translational applications of ASCs for bone regeneration.

Human ASCs isolated from lean and obese donors were characterized based on osteogenic differentiation in adherent cultures and on scaffolds. Critical size calvarial defects were generated in nude mice and treated with scaffolds seeded with ASC isolated from lean or obese donors. Both in vitro and in vivo analysis demonstrated the reduced osteogenic differentiation of ASCs from obese donors, which corresponded to changes in the gene expression profile. Collectively, these findings indicate that obesity significant reduces the osteogenic capacity of ASCs and that ASCs isolated from obese donors may be suboptimal as the cell source for tissue engineering applications.

## Methods

### Human subjects

Primary human ASCs were obtained from subcutaneous abdominal adipose tissue of 12 Caucasian females undergoing elective liposuction. Tissues were obtained with written informed consent under a protocol reviewed and approved by the Pennington Biomedical (Baton Rouge, LA) Institutional Review Board. Lipoaspirates were processed as previously described [31]. The mean BMI for the ASCs isolated from donors with a BMI between 20 and 24.9 (lnASCs) was 22.7 ± 1.9 (N = 6 donors), while the mean BMI for the ASCs isolated from donors with a BMI greater than 30 (obASCs) was 32.7 ± 3.7 (N = 6 donors). The mean age of the subjects for each group of donors was as follows: lnASCs (38.8 ± 7.0) and obASCs (42.5 ± 8.9). No statistical significance in age was observed between the donor groups. Cells were characterized as previously described by our group [30–33].

### Cell culture

Frozen vials of approximately 10^6^ ASCs were thawed, plated onto 150 cm^2^ culture dishes (Nunc, Rochester, NY) in 20 ml CCM and incubated at 37 °C with 5 % humidified CO_2_. After 24 h, medium was removed and adherent, viable cells were washed with PBS, harvested with 0.25 % trypsin/1 mM EDTA (Gibco; Grand Island, NY), and expanded in CCM, which consisted of α-Minimal Essential Medium (Gibco), 20 % fetal bovine serum (FBS; Atlanta Biologicals, Lawrenceville, GA), 100 units per ml penicillin/100 µg/mL streptomycin (P/S; Gibco), and 2 mM l-glutamine (Gibco). Medium was replaced every 3–4 days. For all experiments, cells between passages 2–6 were used.

### Osteogenic differentiation of monolayer cultures

ASCs were cultured in six-well plates in CCM until 70 % confluence. Medium was replaced with fresh osteogenic differentiation medium (ODM) consisting of CCM supplemented with 50 μM ascorbate 2-phosphate (Sigma, St. Louis, MO), 10 mM β-glycerol phosphate (Sigma), and 10 nM dexamethasone (Sigma). Where indicated, the FBS in ODM was substituted for charcoal dextran stripped fetal bovine serum (CDS-ODM; Atlanta Biologicals) and supplemented with log fold increases of 17β-estradiol (E_2_) from 100 pM to 10 μM of estradiol. After 14 or 21 days, cells undergoing osteogenic differentiation in ODM or CDS-ODM were fixed in 10 % formalin for 1 h, washed with distilled water, and stained with 1 % Alizarin Red (pH 4.1) to visualize calcium deposition in the extracellular matrix as a marker of early osteogenesis. Images were acquired at 4 × magnification on Eclipse TE200 (Nikon; Melville, NY) with a digital camera (Nikon DXM1200F) using ACT-1 software (Nikon). For quantification, Alizarin Red was extracted from each well with 10 % cetylpyridinium chloride (CPC) and read at 584 nm (FLUOstar optima). To normalize to the amount of protein in each sample, protein extraction with RIPA buffer (Pierce, Thermo Scientific) and protein quantification with the BCA assay (Thermo Scientific) was performed according to manufacturer’s instructions.

### PLGA scaffold preparation

PLGA scaffolds were fabricated from 85/15 PLGA by solvent casting and lyophilization process, as previously described [34]. The mixture was poured into a polydimethylsiloxane mold (Dow Corning; Midland, MI) to generate 4 mm diameter scaffolds and immediately transferred to a −20 °C freezer. Once the mixture solidified, the scaffolds were immediately freeze-dried for 4 h and sterilized in 70 % ethanol prior to experimentation.

### Cell seeding on PLGA scaffolds

Scaffolds were soaked in PBS for 1 h prior to cell seeding to remove residual ethanol during the sterilization process. Equal number of ASCs from all ASC donors (N = 6) were pooled and directly loaded onto a single face of each scaffold at a concentration of 6000 cells per μl for a total volume of 25 μl for 30 min. The scaffolds were subsequently submerged in 100 μl of growth medium overnight at 37 °C with 5 % humidified CO_2_.

### Osteogenic differentiation of ASCs seeded on PLGA scaffolds

To assess osteogenic differentiation of ASCs seeded on PLGA scaffolds, scaffolds were cultured for 7 days in CDS-CCM or CDS-ODM. Where indicated, medium was supplemented with vehicle, log fold increases of 17β-estradiol from 100 pM to 10 μM. After 7 days, scaffolds were rinsed with PBS, fixed in 70 % ethanol, and stained with 40 mM Alizarin Red (pH 4.1) to visualize calcium deposition. For quantification, Alizarin Red was eluted from each scaffold with 10 % CPC and optical density was read at 584 nm (FLUOstar optima; BMG Labtech, Cary, NC).

### Critical size calvarial defect model

All procedures involving animals were conducted in compliance with State and Federal laws, standards of the US Department of Health and Human Services, and guidelines established by Tulane University Institutional Animal Care and Use Committee (IACUC). All protocols were reviewed and approved by the IACUC prior to implementation. Male Crl:NU-Foxn1^nu^ CD-1 nude mice (60 days old) were obtained from Harlan Laboratories (Indianapolis, IN). After anesthesia, the surgical site was cleaned with povidone/iodine solution (Thermo Scientific) and an incision was made just off the sagittal midline. Periosteum over the region of the incision was incised and lateralized. A unilaterial 4 mm full thickness critical size calvarial defect was created using diamond-coated trephine bits in the nonsuture associated portion of the right parietal bone, taking extreme care to avoid dural injury.

Mice were divided into four treatment groups (N = 6 per group): no scaffold (empty); scaffold alone, lnASC-seeded scaffold, or obASC-seeded scaffold. Following implantation, the skin was sutured and animals were monitored post-operatively. After 16 weeks, animals (N = 3 per time point) were euthanized by cervical dislocation after exposure to CO_2_. Organs were removed and fixed in 10 % formalin for additional analyses.

### Micro-computed tomography (microCT)

Following fixation, the dorsal half of the cranium was scanned (Scanco model 40; Scanco Medical AG, Basserdorf, Switzerland) in a dorsal plane in fluid (70 % ETOH) at 55 kV, 0.3-s integration time, with a 15 µm voxel size and 15 µm slice thickness. The region of interest was defined as a 4 mm-diameter core including the defect. Bone regeneration was determined by bone volume relative to total volume for each group and normalized to the calvarial defects that were left untreated using standard nomenclature [35].

### Histological analysis

Formalin fixed calvaria were decalcified in 10 % EDTA (Sigma), paraffin embedded, and sectioned. Tissue sections stained with hematoxylin and eosin (Richard-Allan Scientific, Thermo Scientific; Waltham, MA), Aniline Blue (Sigma), or Tartrate-Resistant Acid Phosphate (TRAP; Sigma). After staining, slides were dehydrated in graded solutions of ethanol and Sub-X in the final step, and sealed with Permount Mounting Medium (Sigma). Images were acquired and quantified with the ScanScope CS2 (Aperio, Vista, CA) running Image Scope (Aperio).

### RNA isolation, cDNA synthesis, quantitative RT-PCR

ASCs cultured in CDS-ODM for 1, 7, 14 and 21 days were collected, and total cellular RNA was extracted from ASCs using the RNeasy Mini Kit (Qiagen, Valencia, CA), purified with DNase I digestion (Invitrogen) according to manufacturer’s instructions, and reverse transcribed using the SuperScript VILO cDNA synthesis kit (Invitrogen). Quantitative real-time PCR (qRT-PCR) was performed using the EXPRESS SYBR GreenER qPCR SuperMix Kit (Invitrogen) according to the manufacturer’s instructions. The following forward and reverse primer sequences were used to detect changes in gene expression: sp7, 5′-CCAGTGTCTACACCTCTC-3′ and 5′-ATGGAGTAGGAGTGTTGC-3′; runt-related transcription factor 2 (RUNX2), 5′-CTCACTACCACACCTACCTG-3′ and 5′-TCAATATGGTCGCCAAACAGATTC-3′; alkaline phosphatase (ALP), 5′-CTAACTCCTTAGTGCCAGAG-3′ and 5′-CATGATGACATTCTTAGCCAC-3′; FBJ murine osteosarcoma viral oncogene homolog (c-Fos), 5′-CCTGTCAAGAGCATCAGCAG-3′ and 5′-GTCAGAGGAAGGCTCATTGC-5′; distal-less homeobox 5 (DLX5), 5′-TGGCCCGAGTCTTCAGCTAC’ and 5′-TGGTTGGTCGGTCTCTTTCT-3′; osteonectin (SPARC), 5′-TGTGGGAGCTAATCCTGTCC-3′ and 5′-TCAGGACGTTCTTGAGCCAGT-3′; osteopontin (OPN), 5′-GCTCTAGAATGAGAATTGCACTG-3′ and 5′-GTCAATGGAGTCCTGGCTGT-3′; collagen type 1a (COLA1a), 5′-CATGTTCAGCTTTGTGGACCTC-3′ and 5′-AGGTGATTGGTGGGATGTCTT-3′; insulin-like growth factor 1(IGF1), 5′-CTGTGATCTAAGGAGGCTG-3′ and 5′-TTCGTGTTCTTGTTGGTAGA-3′; and beta-actin (β-actin), 5′-CACCTTCTACAATGAGCTGC-3′ and 3′-TCTTCTCGATGCTCGACGGA-3′. All RT-PCR primers were designed using Primer3 (Boston, MA) and purchased from Integrated DNA Technologies (Coralville, IA). The expression of human β-actin was used to normalize mRNA content. Samples were tested in triplicates. No-template controls and no-reverse transcription controls were included in each PCR run. Data was normalized to lnASCs exposed to CDS-ODM for 1 day to account for differences associated with serum.

### Statistical analysis

All values are presented as mean ± standard error of the means (SEM). The statistical differences between two or more groups were determined by ANOVA, followed by post hoc Tukey multiple comparison tests. Statistical significant was set at *P* < 0.05. Only statistically significant values were considered relevant. Analysis was performed using Prism (Graphpad Software, San Diego, CA).

## Results

### obASCs demonstrate reduced osteogenic differentiation potential in a growth factor depleted environment

**Fig. 1:**
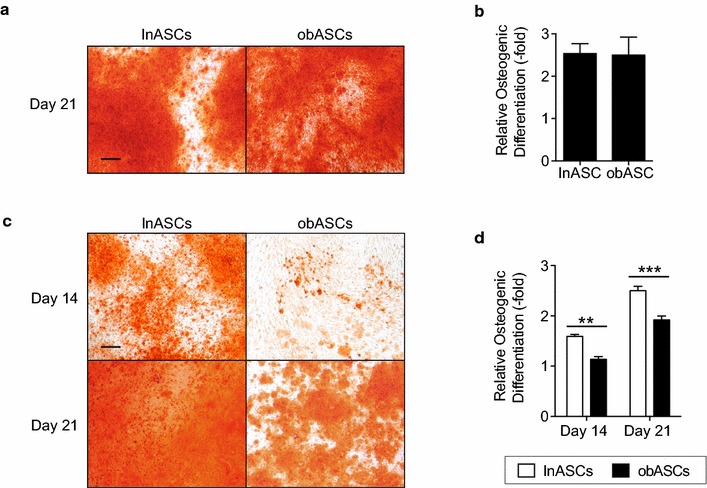
obASCs display reduced osteogenic differentiation in an estrogen depleted environment. **a** lnASCs (N = 6 donors) and obASCs (N = 6 donors) were cultured in ODM and stained with alizarin red after 21 days for calcium deposition. **b** To quantify the amount of alizarin red staining, stains were eluted with CPC and optical density was read. **c** lnASCs (N = 6 donors) and obASCs (N = 6 donors) were cultured in CDS-ODM and stained with alizarin red after 14 and 21 days for calcium deposition. **d** To quantify the amount of alizarin red staining, stains were eluted with CPC and read at 590 nm. *Scale bar* represents 200 µm. Bars, ± SEM. **, *P* < 0.01; ***, *P* < 0.001

To characterize the osteogenic potential of lnASCs and obASCs, these cells were induced with ODM for 21 days and calcium deposition was visualized with Alizarin Red staining. No statistically significant difference was observed between lnASCs and obASCs cultured in ODM (Fig. 1a, b). However, when lnASCs and obASCs were cultured in CDS-ODM, visible differences in the amount of calcium deposition were apparent (Fig. 1c). lnASCs demonstrated a 1.6-fold and a 2.5-fold increase in osteogenic differentiation after 14 and 21 days, respectively, while obASCs demonstrated a 1.1-fold and a 1.9-fold increase in osteogenic differentiation after 14 and 21 days, respectively (P < 0.01; Fig. 1d). These findings suggest that obASCs have a diminished osteogenic capacity in the absence of estrogens. To determine whether delivery of estradiol would restore the osteogenic differentiation in obASCs, cells were induced with CDS-ODM supplemented with log fold increases of estradiol from 100 pM to 10 μM. Both lnASCs and obASCs demonstrated enhanced osteogenic differentiation in the presence of estradiol (Additional file 1: Fig. S1). Since delivery of estradiol restored the osteogenic differentiation capacity of obASCs, these results indicate that estradiol, in part, is a necessary competent for osteogenic differentiation of obASCs (Additional file 1: Fig. S1).

### obASCs display reduced osteogenic differentiation capacity on PLGA scaffolds

**Fig. 2:**
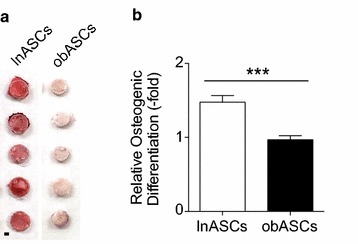
obASCs seeded on scaffolds display reduced osteogenic differentiation compared to lnASCs. lnASCs and obASCs were seeded on PLGA scaffolds and induced with CDS-ODM for 7 days. **a** Scaffolds stained with Alizarin Red are shown. **b** Stains were eluted from scaffolds with CPC and quantified. *Scale bar* represents 1 mm. Bars, ± SEM. ***, *P* < 0.001

To investigate the osteogenic differentiation potential of lnASCs and obASCs on a biodegradable scaffold, lnASCs and obASCs were seeded on a PLGA scaffold cultured in CDS-CCM and analyzed by Alizarin Red staining. After 7 days, lnASCs seeded on scaffolds displayed enhanced osteogenesis to 1.5-fold, meanwhile obASCs seeded on scaffolds did not demonstrate similar osteogenic effects (P < 0.05, Fig. 2). Supplementation with estradiol restored the osteogenic capacity of obASCs cultured in CDS-ODM, indicating that obASCs require estradiol for differentiation (Additional file 2: Fig. S2).

### obASCs was unable to induce osteogenesis activity in vivo compared to lnASCs

**Fig. 3:**
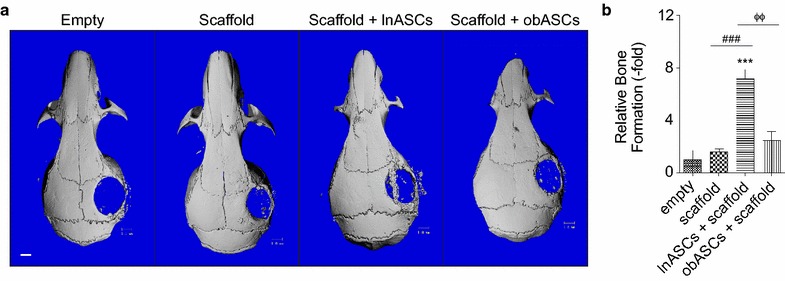
obASCs seeded on scaffolds display reduced osteogenesis in vivo. Critical size calvarial defects were created in the right parietal bone of nude mice. Treatment groups included defects without scaffold or cells (empty), defects with scaffold only and defects treated scaffolds seeded with lnASCs or obASCs. **a** Representative 3D reconstructions of microCT scans performed at 16 weeks. **b** Quantification of microCT. *Scale bar* represents 1 mm. Bars, ± SEM. ***, P < 0.001, compared to untreated defects; ^###^, P < 0.001, compared between defects treated with scaffold and scaffolds seeded with lnASCs; ^ΦΦ^, P < 0.01, compared between defects treated with scaffolds seeded with lnASCs and obASCs

**Fig. 4:**
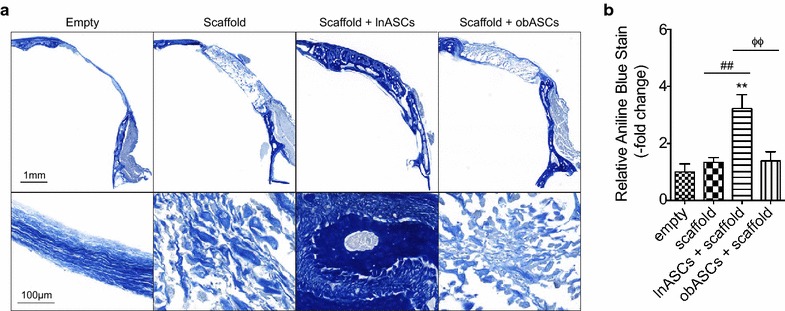
Defects treated with lnASCs-seeded scaffolds demonstrate significant collagen deposition. Calvarial defects were created in the parietal bone of nude mice. Histology was conducted 16 weeks post operatively. **a** Representative images of sections stained with aniline blue are shown. **b** Quantification of collagen deposition. *Bars*, ± SEM. **, P < 0.01, compared to untreated defects; ^##^, P < 0.01, compared between defects treated with scaffold and scaffolds seeded with lnASCs. ^ΦΦ^, P < 0.01, compared between defects treated with scaffolds seeded with lnASCs and obASCs

To assess osteogenesis of lnASCs and obASCs on PLGA scaffolds in vivo, critical size calvarial defects were created in nude mice and left empty or treated with scaffolds, lnASC-seeded scaffolds, or obASC-seeded scaffolds. MicroCT was performed on the skulls 2 and 16 weeks after implantation to visualize and quantify bone formation. At 2 weeks, no statistically significant difference was observed between any of the groups, which was expected given the lack of calcified bone at such an early time point (Additional file 3: Fig. S3). By 16 weeks, mice treated with scaffolds generated more calcified regions (2.1-fold increase) compared to mice in the control group; however, these results were not statistically significant (Fig. 3). The addition of lnASCs to scaffolds enhanced osteogenesis to 7.2-fold; meanwhile, the addition of obASCs only induced a 2.5-fold increase in calcified bone (P < 0.05, Fig. 3). Histological analysis revealed significantly higher levels of collagen, visualized by Aniline Blue staining. Consistent with the microCT analysis, mice treated with scaffolds alone demonstrated higher levels of collagen, compared to mice without treatment (P < 0.05; Fig. 4). Mice treated with lnASCs demonstrated significantly higher levels of collagen deposition (3.2-fold), compared to mice treated with scaffold alone (1.3-fold) or obASC-seeded scaffolds (1.4-fold; P < 0.05; Fig. 4). Aniline blue demonstrated complete coverage of the defect, though this tissue had not completely ossified by microCT analysis. These results suggest that obASCs were unable to induce osteogenesis within scaffolds in vivo, indicating that obASCs may have reduced osteogenic properties compared to lnASCs.

### Calvarial defects treated with lnASCs or obASCs displayed reduced osteoclast activity

**Fig. 5:**
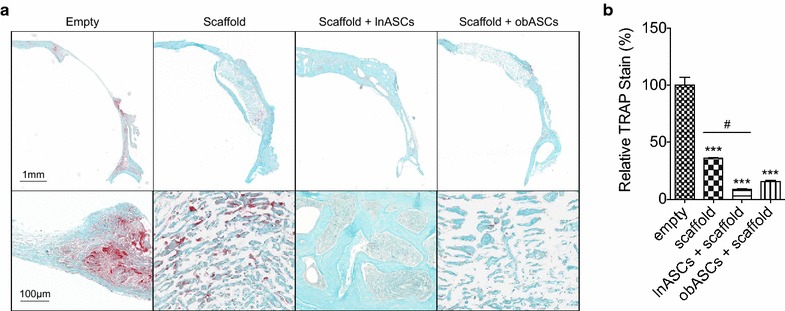
Calvarial defects treated with lnASC- or obASCs-seeded scaffolds display reduced osteoclastic activity. Calvarial defects were created in the parietal bone of nude mice. Histology was conducted 2 weeks post operatively. **a** Representative images of sections stained with TRAP, allowing osteoclasts to appear bright red. **b** Quantification of TRAP stain. Bars, ± SEM. ***, P < 0.001, compared to defects treated with neither scaffolds or cells; ^#^, P < 0.05, compared between defects treated with scaffold and scaffolds seeded with lnASCs

While ASCs have previously been shown to enhance osteogenesis through osteoblastogenesis, it is possible that ASCs may generate an environment that inhibits osteoclastogenesis to reduce the breakdown of bone. To assess whether lnASCs and obASCs within scaffolds altered osteoclast activity during early remodeling, skull sections were stained with TRAP and assessed quantitatively. At 2 weeks, defects implanted with scaffold alone demonstrated reduced osteoclast activity compared to those left untreated (Fig. 5). Defects treated with obASC within scaffolds demonstrated a reduction in osteoclast activity to 15.8 %; meanwhile, lnASCs within scaffolds demonstrated even less osteoclast activity with reduction to 8.7 % (Fig. 5). These results indicate that lnASCs provide an environment to support osteogenesis while inhibiting osteoclastogenesis.

### obASCs display an altered osteogenic gene expression profile compared to lnASCs

**Fig. 6:**
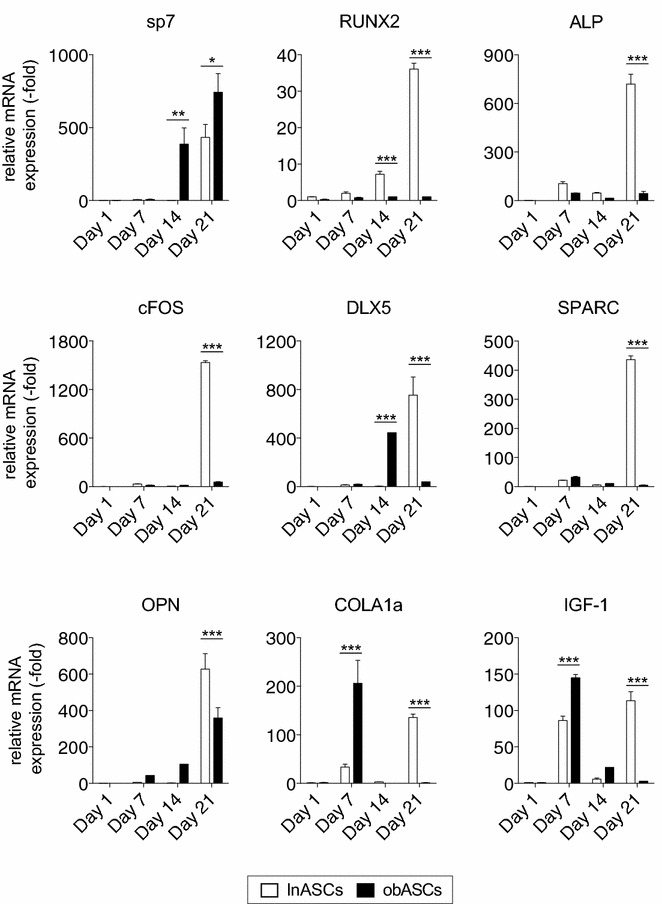
The gene expression pattern of lnASCs and obASCs differ during osteogenic differentiation. lnASCs (N = 6 donors) and obASCs (N = 6 donors) were cultured in CDS-ODM and harvested after 1, 7, 14, and 21 days for qRT-PCR analysis of key osteogenic factors. Data is normalized to lnASCs cultured in CDS-ODM for 1 day, set to 1.0. *Bars*, ± SEM. *, P < 0.05; **, P < 0.01; ***, P < 0.001 between lnASCs and obASCs

To further investigate the reduced osteogenic differentiation capacity of obASCs, lnASCs and obASCs were induced with CDS-ODM for 1, 7, 14, and 21 days, and the mRNA expression of osteogenic genes was assessed. lnASCs exposed to CDS-ODM for 1 day was chosen as the baseline control to compare across different time points and different cell types. lnASCs induced with CDS-ODM demonstrated the most robust induction of osteogenic genes after 21 days in Sp7 (434.0-fold), RUNX2 (36.1-fold), ALP (720.5-fold), c-FOS (1534.1-fold), DLX5 (753.8-fold), SPARC (436.5-fold), and OPN (627.6-fold), compared to lnASCs exposed to CDS-ODM for 1 day (Fig. 6). In contrast, obASCs induced with CDS-ODM did not demonstrate upregulation of most of these osteogenic genes; osteogenic genes RUNX2, ALP, cFOS, and SPARC were not upregulated in obASCs following induction during any of the time points. Only Sp7 was upregulated in obASCs after 14 days (387.4-fold) and 21 days (743.9-fold) CDS-ODM, and a 444.9-fold increase in DLX5 expression was observed in obASCs after 14 days in CDS-ODM. Furthermore, lnASCs cultured in CDS-ODM demonstrated a biphasic upregulation of COLA1a by 33.6-fold and 135.7-fold after 7 and 21 days, respectively (Fig. 6). lnASCs induced with CDS-ODM displayed a biphasic pattern of expression of IGF-1, with a 86.2-fold and 113.5-fold induction in lnASCs after 7 days and 21 days in CDS-ODM (Fig. 6). In contrast, obASCs demonstrated a robust upregulation of COLA1a by 206.0-fold and IGF1 by 145.0-fold after 7 days in CDS-ODM, without the biphasic induction after 21 days (Fig. 6). These findings together suggest that while lnASCs temporally upregulate osteogenic genes during early, mid, and late osteogenic differentiation, obASCs demonstrate diminished capacity to upregulate key osteogenic genes at specific time points during differentiation.

## Discussion

Bone tissue engineering is an interdisciplinary field focused on the development of biological bone substitutes that restore, maintain, or improve tissue function by applying the principles of engineering and life sciences [36]. The primary target of clinical therapeutic strategies is the regeneration of bone for structural reconstruction. Strategies aimed at stimulating bone healing to treat skeletal defects are pertinent due to the large number of bone grafting procedures performed annually worldwide [37, 38].

Advances in utilizing sophisticated bone scaffolds eliminate the risk of disease transmission, reduce the number of surgical procedures performed, and reduce the risk of infection or immunogenicity [39]. These bone scaffolds provide an endless supply of synthetic and natural biomaterials, eliminating the need to center surgical procedures on available autologous bone. Lately, the addition of ASCs to the tissue engineering strategy has gained significant interest and attention. The synergistic effects of biodegradable scaffolds and ASCs enhance bone formation by allowing ASCs to engraft and undergo osteogenic differentiation [40]. The restorable nature of these constructs also allows for the integration with the surrounding tissue and eventual replacement by new or existing host tissue [40]. Sándor demonstrated the synergistic effect of ASCs, restorable scaffold, and growth factor in patients with craniofacial osseous defects, whereby delivery of tissue engineered constructs expedited bone regeneration at the site of implantation [41]. Additional large-scale studies are necessary to confirm the reproducibility of this technique in a large cohort, but these preliminary findings are particularly promising. Furthermore, it is unclear if the viability of the obASCs differs from the lnASCs and how long these cells persist in vivo. Clinically, the improved bone formation in the lnASCs indicates that diabetic patients might benefit from lnASCs when faced with a non-healing bone defect. Alternatively, the obASCs might require implantation with additional osteogenic growth factors.

To ensure the successful regeneration of bone with ASCs as the cell source, studies investigating the ideal donor to harvest ASCs from are necessary. In the current study, the impact of obesity on the bone regenerative potential of lnASCs and obASCs was investigated. Consistent with previous reports [42, 43], the addition of lnASCs enhanced the osteoinductive effects of scaffolds both in vitro and in vivo, compared to the untreated control groups and groups treated with scaffold alone. Scaffolds seeded with obASCs demonstrated similar findings as scaffold alone, suggesting the addition of obASCs has no impact on bone regeneration. During the osteogenic differentiation of lnASCs, several transcription factors were notably upregulated, including RUNX2, ALP, cFOS, DLX5, SPARC, OPN, COLA1a, and IGF-1. RUNX2 is considered a central control gene within the osteoblast lineage, thereby enabling RUNX2 to directly stimulate downstream ALP, osteocalcin, OPN, COLA1a, bone sialoprotein, and RANKL [44, 45]. It is the coordinated upregulation of these osteogenic factors that leads to the differentiation of cells into osteoblasts and result in the deposit of extracellular matrix proteins that regenerates bone [44, 45].

In contrast to the robust osteogenic differentiation of lnASCs, obASCs in the present study displayed reduced osteogenic differentiation in adherent cultures, on scaffolds in culture, and on scaffolds in a critical size calvarial defect model. The reduced osteogenic differentiation correlated with reduced collagen deposition. These results would suggest that the osteoinductive effects of obASCs are attenuated compared to lnASCs. Studies have shown that obesity increases the presence of proinflammatory cytokines in circulation and in tissues, which may alter the osteogenic differentiation of ASCs and promote osteoclast activity and bone resorption [46–49]. Cao et al. further demonstrated that high fat diet decreased calcium availability for bone formation [48]. Together, these altered processes counteract the traditional thought that obesity due to mechanical load may be beneficial to bone [50].

The data demonstrate that the delivery of estradiol rescued the effects of obesity-related changes to osteogenic differentiation. This finding indicates that the high levels of estradiol associated with obesity may alter the biology of ASCs such that estradiol is now essential for the maintenance of these cells. These results indicate that obesity may directly or indirectly alter ASC biology and lead to permanent imprinting on ASCs. With respect to tissue engineering, obesity appears to reduce the osteoinductive effects of ASCs and may reduce the efficacy of engineered constructs generated with obASCs as the cell source.

Mechanistically, the expression of key osteogenic genes was attenuated in obASCs during osteogenic differentiation, compared to the expression pattern in lnASCs. obASCs displayed minimal RUNX2, ALP, cFOS, and SPARC expression. Interestingly, the levels of COLA1a, IGF-1, DLX5 and sp7 induction were temporally and spatially enhanced in obASCs, compared to lnASCs. It should be noted that while lnASCs display a biphasic upregulation of COLA1a and IGF-1, obASCs display a more robust response during early osteogenic differentiation with no induction during late osteogenic differentiation. As markers of late osteogenesis, the lack of COLA1a and IGF-1 in obASCs during late osteogenesis would suggest that obesity has altered osteogenic differentiation of these cells mechanistically. The robust upregulation COLA1a and IGF-1 during early osteogenic differentiation is likely due to the activation of alternative osteogenic pathways. Previous studies have shown that IGF-1 induces Dlx5, which in turn has been shown to induce sp7 expression [51, 52]. Studies have shown that sp7 acts in the regulation of numerous osteoblast genes including osteocalcin, SPARC, OPN, bone sialoprotein, and COLA1a [53]. Consistent with these previous reports, obesity may have a role in inhibiting downstream regulatory activity of sp7 and reducing the ability of sp7 to induce osteonectin and COLA1a expression. Together, these changes in the gene expression profile of obASCs during osteogenic differentiation, compared to lnASCs, are consistent with the poor osteogenic differentiation of these cells and highlight the underlying alterations to ASC osteogenic differentiation.

## Conclusion

Collectively, the findings from this study would suggest that obASCs have diminished osteogenic differentiation capacity and may be suboptimal as the cellular component to tissue engineering approaches. Future studies are necessary to determine how obesity alters ASC biology. It is unknown whether it is the long-term direct contact with dysregulated adipose tissue or the exposure to the secretome of adipose tissue that alters ASCs. Additional studies are necessary to identify the pathways altered by obesity in ASCs in order to reverse the effects of obesity on ASC osteogenesis.

## Additional files

10.1186/s12967-016-0776-1 Estradiol restores the osteogenic differentiation capacity of obASCs. (A) lnASCs (n=6 donors) and obASCs (n=6 donors) were cultured in CDS-ODM supplemented with estradiol. After 14 days, cells were stained with Alizarin Red. Scale bar represents 200 μm. (B) To quantify the amount of Alizarin Red staining, stains were eluted with CPC and optical density was read. Bars, ± SEM. *, P < 0.05; **, P < 0.01 between lnASCs and obASCs.
10.1186/s12967-016-0776-1 Estradiol enhanced osteogenic differentiation of obASCs. lnASCs and obASCs were seeded on PLGA scaffolds and induced with CDS-ODM supplemented with 10 Nm estradiol. Scaffolds stained with Alizarin Red are shown. Scale bar represents 1 mm.
10.1186/s12967-016-0776-1 No observable differences in lnASCs and obASCs during early bone regeneration. Critical size calvarial defects were created in the parietal bone of nude mice and assessed after 2 weeks. (A) Representative images of microCT scanning. (B) Quantification of microCT. Scale bar represents 1 mm. Bars, ± SEM.
