# Supplementary figures and images for: Obesity inhibits the osteogenic differentiation of human adipose-derived stem cells
Source: J Transl Med. 2016 Jan 27;14:27. doi: 10.1186/s12967-016-0776-1 (PMC4730660; doi:10.1186/s12967-016-0776-1)

A

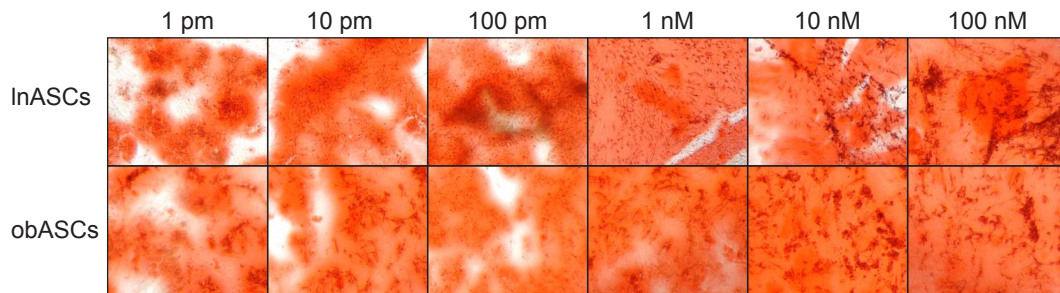

B

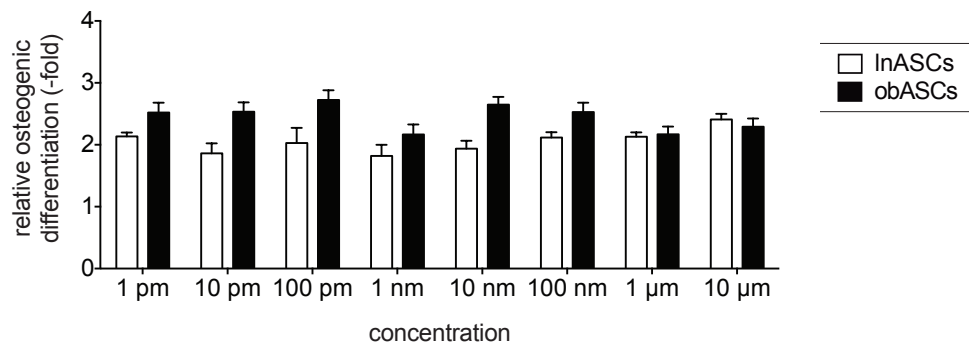

Supplement: Supplementary file 1 — 10.1186/s12967-016-0776-1 Estradiol restores the osteogenic differentiation capacity of obASCs. (A) lnASCs (n=6 donors) and obASCs (n=6 donors) were cultured in CDS-ODM supplemented with estradiol. After 14 days, cells were stained with Alizarin Red. Scale bar represents 200 μm. (B) To quantify the amount of Alizarin Red staining, stains were eluted with CPC and optical density was read. Bars, ± SEM. *, P < 0.05; **, P < 0.01 between lnASCs and obASCs. [file 12967_2016_776_MOESM1_ESM.pdf]

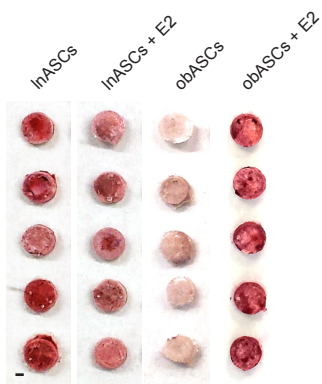

Supplement: Supplementary file 2 — 10.1186/s12967-016-0776-1 Estradiol enhanced osteogenic differentiation of obASCs. lnASCs and obASCs were seeded on PLGA scaffolds and induced with CDS-ODM supplemented with 10 Nm estradiol. Scaffolds stained with Alizarin Red are shown. Scale bar represents 1 mm. [file 12967_2016_776_MOESM2_ESM.pdf]

A

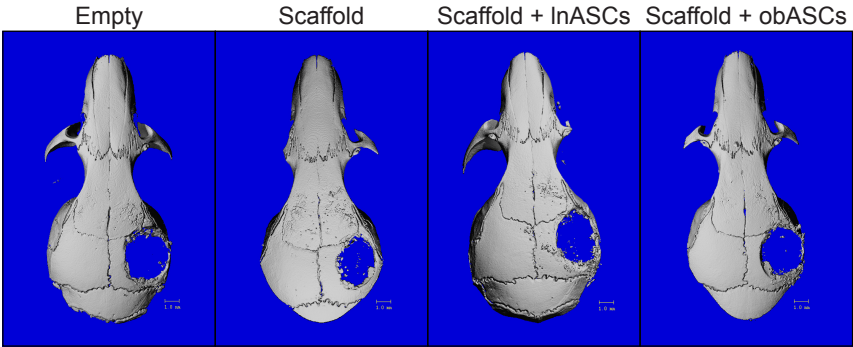

B

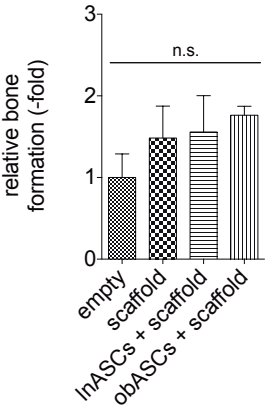

Supplement: Supplementary file 3 — 10.1186/s12967-016-0776-1 No observable differences in lnASCs and obASCs during early bone regeneration. Critical size calvarial defects were created in the parietal bone of nude mice and assessed after 2 weeks. (A) Representative images of microCT scanning. (B) Quantification of microCT. Scale bar represents 1 mm. Bars, ± SEM. [file 12967_2016_776_MOESM3_ESM.pdf]
